# Supplementary material for: An Integrative Pancreatic Cancer Risk Prediction Model in the UK Biobank
Source: Biomedicines. 2023 Dec 1;11(12):3206. doi: 10.3390/biomedicines11123206 (PMC10740416; doi:10.3390/biomedicines11123206)
Supplement: Supplementary file 1 [file biomedicines-11-03206-s001.zip › biomedicines-2733494-supplementary.pdf]

Supplementary Content

Table S1. Codes used to identify pancreatic cancer (PaCa) cases and cancer-free controls in the UK Biobank cohort.

Table S2. Classification of the exposure variables included in the analysis.

Table S3. 96 single nucleotide polymorphisms (SNPs) extracted from the UK Biobank cohort.

Table S4. 40 single nucleotide polymorphisms (SNPs) were used in calculating the PaCa polygenic risk scores (PRS) in the UK Biobank study.

Table S5. Missing Rate of four variables.

Table S6. Demographic characteristics of pancreatic cancer (PaCa) were analyzed across different case and cancer-free control ratio groups.

Table S7. Confusion matrix by 10-fold cross-validation method in the training dataset and testing dataset.

Table S8. Pancreatic cancer (PaCa) risk factors in the multivariable logistic regression model across different case and cancer-free control ratio groups.

Table S9. Bootstrap 500 repetitions for internal validation of the model performance.

Table S10. The equation for constructing the nomogram.

Figure S1. The flowchart of PaCa cases and cancer-free controls selection process in the UKB cohort.

Figure S2. The flowchart of single nucleotide polymorphisms (SNPs) extraction, SNPs quality control (QC), Sample QC and PRS construction.

Figure S3. The SHAP summary plot

Figure S4. SHAP waterfall plot

Figure S5. The receiver operating characteristic (ROC) curve for the random forest. (a) The receiver operating characteristic (ROC) curve for the random forest in the 85% training dataset. (b) The receiver operating characteristic (ROC) curve for the random forest in the 15% testing dataset. (c) The receiver operating characteristic (ROC) curve for the random forest in the 70% training dataset. (d) The receiver operating characteristic (ROC) curve for the random forest in the 30% testing dataset.

Figure S6. The importance of each variable in the sensitivity test.

Figure S7. The receiver operating characteristic curve (ROC) for whole logistic regression and without PRS model.

Figure S8. A nomogram based on the multivariable logistic model.

Figure S9. A dynamic nomogram based on the multivariable logistic model.

Table S1. Codes used to identify pancreatic cancer (PaCa) cases and cancer-free controls in the UK Biobank cohort

**Sources**	**Pancreatic cancer cases**	**Cancer-free controls**	**Subjects excluded from the study**	
	Incident cases	All healthy controls	Other neoplasms (including in situ neoplasms, benign neoplasms and neoplasms of unknown nature or behaviour)	Prevalent cases
**ICD10 codes**	Codes start with C25 and its subclasses, C25.0, C25.1, C25.2, C25.3, C25.4, C25.7, C25.8, and C25.9	Subjects with no code assigned	Codes start with C (C00-C97) except codes for PaCa, and codes begin with D (D00-D48)	Codes start with C25 and its subclasses, C25.0, C25.1, C25.2, C25.3, C25.4, C25.7, C25.8, and C25.9
**ICD9 codes**	Codes start with 157 and its subclasses 1570, 1574, and 1579	Subjects with no code assigned	Codes 140-239, except codes for PaCa	Codes begin with 157 and its subclasses 1570, 1574, and 1579
**Self-reported cancer codes**	1026 code only	Subjects with no code assigned	All other codes except the 1026 code	1026 code only
**Frequency (%)**	1,402 (0.28%)	389,027 (77.44%)	111,866 (22.27%)	92 (0.02%)

Table S2. Classification of the exposure variables included in the analysis.

	Variable	Groups	Coding
1.	Gender	Female	This variable is described as the biological sex provided by the UK Biobank (reference field ID: 31).
		Male	
2.	Age	Continuous data	The age is described as the age when attending the assessment centre provided by the UK Biobank (reference field ID:21003).
3.	Blood type	O blood type	This variable is described as a blood-type haplotype provided by the UK Biobank (reference field ID: 23165). Data coding AA, AB, AO, BB and BO were categorised as Non-O blood types. Data coding OO was classified as O blood type.
		Non-O blood type	
4.	Family history of bowel cancer	Yes or No	This variable is described as illnesses of the father and mother provided by the UK Biobank (reference field ID: 20107 and 20110)
5.	Polygenic Score (PRS)	Q1	This variable is described as imputation from genotype (WTCHG) by the UK Biobank (reference field ID: 22828). All the processes of single nucleotide polymorphisms (SNPs) extraction, SNPs quality control (QC), sample QC and PRS construction are described in the supplement figure S1, Table S3 and S4.
		Q2	
		Q3	
		Q4	
		Q5	
6.	Tobacco smoking (including cigarettes, pipes, cigars, etc.)	Never	This variable is described as the smoking status information provided by the UK Biobank (reference field ID: 20116).
		Previous	
		Current	
7.	Alcohol intake amount	Never	The alcohol drinker status computes this variable, alcohol intake frequency, average weekly red wine intake, average monthly red wine intake, average weekly champagne plus white wine intake, average monthly champagne plus white wine intake, average weekly beer plus cider intake, average monthly beer plus cider intake, average weekly fortified wine intake, average monthly fortified wine intake, average weekly spirits intake, average monthly spirits intake, average weekly intake of other alcoholic drinks and average monthly intake of different alcoholic drinks provided by the UK Biobank (reference field ID: 20117, 1558, 1568, 4407,1578, 4418, 1588, 4429, 1608, 4451, 1598, 4440,5364, and 4462). The quantity of each type of drink (red wine, white wine, beer or cider, fortified wine or spirits) was multiplied by its standard drink size and reference alcohol content. The alcohol drinking frequency was summed and converted to the g/day alcohol intake amount. One unit equals 10ml or 8g of pure alcohol [30].
		Men: >0-≤28 g/d, Women: >0-≤14 g/d	
		Men: >28 g/d, Women: >14 g/d	

	Variable	Groups	Coding
			The category of alcohol intake amount was adapted from the 2018 World Cancer Research Fund/American Institute for Cancer Research (WCRF/AICR) Cancer Prevention Recommendations [28].
8.	Body mass index (BMI)	Normal or underweight (BMI<25)	The BMI information is continuous data initially provided by the UK Biobank (reference field ID:21001). The classification was divided into three groups based on NHS suggestion [31].
		Overweight (25 ≤ BMI < 30)	
		Obese (BMI ≥ 30)	
9.	Waist Hip Ratio	Normal (M: <0.90, F: <0.85)	This variable is initially calculated by dividing the waist circumference over the hip circumference (reference field ID:49) and then categorised into the normal group and abdominal obesity group according to WHO recommendation [32].
		Abdominal obesity (M: ≥0.90, F: ≥0.85)	
10.	Physical activity, MET-min/wk*	<600 MET-min/wk	This variable is described as the Summed MET minutes per week for all activity provided by the UK Biobank (reference field ID: 22040). The category of MET was adapted from the 2018 World Cancer Research Fund/American Institute for Cancer Research (WCRF/AICR) Cancer Prevention Recommendations [28] and previous other UK biobank study [33].<br>*600 MET-min/wk = 150 min/wk; 3000 MET-min/wk = 750 min/wk.
		600-3000 MET-min/wk	
		>3000 MET-min/wk	
11.	Pancreatitis	Yes or No	This variable is obtained by using:<br>1. Self-reported non-cancer illness medical condition (reference field ID: 20002) to identify pancreatitis code 1165<br>2. Hospital record outcome ICD9 Summary Diagnoses (reference field ID: 41271 and 41203) to identify acute pancreatitis code 5770 and chronic pancreatitis code 5771.<br>3. Hospital record outcome ICD10 Summary Diagnoses (reference field ID: 41280 and 41262) to identify acute pancreatitis code K85 and chronic pancreatitis code K86.<br>4. Health-related outcomes acute pancreatitis report (reference field ID:131682)
12.	Diabetes Mellitus	Yes or No	This variable is obtained by using:<br>1. Self-reported non-cancer illness medical conditions (reference field ID: 20002) to identify Diabetes Mellitus codes 1220,1222 and 1223.<br>2. Hospital record outcome ICD9 Summary Diagnoses (reference field ID: 41271 and 41203) to identify Diabetes Mellitus code 250.

	Variable	Groups	Coding
			3. Hospital record outcome ICD10 Summary Diagnoses (reference field ID: 41280 and 41262) to identify Diabetes Mellitus code E10-E14.<br>4. Health-related outcomes report diabetes mellitus (reference field ID:130706,130708,130710,130712, and 130714)<br>5. Assessment centre medical condition diabetes diagnosed by a doctor (reference field ID: 2443)
13.	Hepatitis B	Yes or No	This variable is obtained by using:<br>1. self-reported non-cancer illness medical condition (reference field ID: 20002) to identify Hepatitis B code 1579.<br>2. Hospital record outcome ICD9 Summary Diagnoses (reference field ID: 41271 and 41203) to identify Hepatitis B code 0703.<br>3. Hospital record outcome ICD10 Summary Diagnoses (reference field ID: 41280 and 41262) to identify Acute hepatitis B code B16, Chronic viral hepatitis B B180-B181.<br>4. HBV seropositivity for Hepatitis B Virus (reference field ID: 23060)<br>5. Health-related outcomes report for acute hepatitis B (reference field ID:130196).
14.	Cholecystitis/Cholelithiasis/ Cholecystectomy	Yes or No	Cholecystitis/Cholelithiasis is obtained by using:<br>1. Self-reported non-cancer illness medical condition (reference field ID: 20002) to identify cholecystitis code 1163 and cholelithiasis/gall stones code 1162.<br>2. Hospital record outcome ICD9 Summary Diagnoses (reference field ID: 41271 and 41203) to identify cholecystitis code 574 and cholelithiasis/gall stones code 5750 and 5751.<br>3. Hospital record outcome ICD10 Summary Diagnoses (reference field ID: 41280 and 41262) to cholecystitis code K81 and cholelithiasis/gall stones code K80.<br>4. Health-related outcomes report for acute cholecystitis (reference field ID:131676) and cholelithiasis (reference field ID:131674).<br>Cholecystectomy is obtained by using:<br>1. Assessment centre operation code for Cholecystectomy (reference field ID: 20004).<br>2. Operative procedures record for Cholecystectomy (reference field ID: 41272 and 41256).
15.	Helicobacter pylori (H.pylori) infection	Yes or No	This variable is obtained by using:

	Variable	Groups	Coding
			1. Self-reported non-cancer illness medical condition (reference field ID: 20002) to identify Helicobacter pylori infection code 1442.<br>2. Hospital record outcome ICD10 Summary Diagnoses (reference field ID: 41280 and 41262) to Helicobacter pylori infection code B980.<br>3. Helicobacter pylori seropositivity (reference field ID: 23073 and 23074).
16.	Systemic lupus erythematosus (SLE)	Yes or No	This variable is obtained by using:<br>1. Self-reported non-cancer illness medical condition (reference field ID: 20002) to identify Systemic lupus erythematosus code 1381.<br>2. Hospital record outcome ICD9 Summary Diagnoses (reference field ID: 41271 and 41203) to SLE code 7100.<br>3. Hospital record outcome ICD10 Summary Diagnoses (reference field ID: 41280 and 41262) to SLE code M32.<br>4. Health-related outcomes report for SLE (reference field ID: 131894).
17.	Periodontal disease	Yes or No	This variable is obtained by using:<br>1. Hospital record outcome ICD9 Summary Diagnoses (reference field ID: 41271 and 41203) to Periodontal disease code 5233, 5234, 5238, and 5239.<br>2. Hospital record outcome ICD10 Summary Diagnoses (reference field ID: 41280 and 41262) to Periodontal disease code K05.2-K05.6.<br>3. Health-related outcomes report for Periodontal disease (reference field ID: 131562).
18.	Vitamin D deficiency	Yes or No	This variable is obtained by using:<br>1. Hospital record outcome ICD10 Summary Diagnoses (reference field ID: 41280 and 41262) to Periodontal disease code E55.

Table S3 96 single nucleotide polymorphisms (SNPs) extracted from the UK Biobank cohort.

CHR	SNP	BP	Risk Allele	P value	OR	RAF	Mapped gene	Reference: PubMed ID
1	rs351365	112503773	C	6 x 10-6	1.11	0.74	WNT2B	29422604(2018)
1	rs13303010	959193	G	8 x 10-14	1.26	0.11	NOC2L	29422604(2018)
1	rs2816938	200016240	A	3 x 10-15	1.21	0.23	RNU6-609P, NR5A2	27579533(2016),29422604(2018)
1	rs3790844	200038304	A	8 x 10-16	1.23	0.77	NR5A2	29422604(2018),26098869(2015), 20101243(2010)
1	rs1747924	64073289	A	7 x 10-6	1.11	0.77	ROR1	26098869(2015)
1	rs1326889	230727252	C	4 x 10-7	1.11	NR	AGT	30541042(2019)
1	rs2689154	238745053	G	6 x 10-6	1.2	0.17	KRT18P32, MIPEPP2	22158540(2011)
1	rs60579835	112570572	T	1 x 10-8	1.48	0.47	ST7L	32514122(2020)
2	rs12478462	152798206	G	3 x 10-7	1.14	0.22	UBQLN4P2, ARL6IP6	29422604(2018)
2	rs1486134	67412637	G	4.61 x 10-9	1.13	0.28	ETAA1, LINC02831	29422604(2018),26098869(2015)
2	rs192904742	6581986	A	9 x 10-7	4.12	NR	MIR7515HG, LINC01824	32887889(2020)
2	rs71411601	52785787	C	2 x 10-7	2.79	0.01	MIR4431, ASB3	32887889(2020)
2	rs962856	67366671	C	2 x 10-8	1.12	0.38	LINC01829	26098869(2015)
2	rs6736997	234706553	A	2.95 x 10-4	1.20	0.33	LINC01173	20686608(2010)
2	rs12615966	104762499	A	7 x 10-6	3.15	0.10	PANTR1, LINC01114	20686608(2010)
2	rs6711606	101305708	A	4 x 10-6	2.81	0.12	RNF149	20686608(2010)
2	rs1427593	136797654	A	7 x 10-6	1.49	0.08	THSD7B	20686608(2010)
2	rs183117027	21004340	A	4 x 10-8	2.34	0.01	APOB	30206226(2018)
3	rs9854771	189790682	G	5 x 10-8	1.11	0.64	TP63	29422604(2018),26098869(2015)
3	rs148512905	18943255	C	1 x 10-7	2.68	0.01	RNU6-138P, SATB1-AS1	32887889(2020)
3	rs138585571	98469736	T	3 x 10-7	1.81	0.04	OR5K1	32887889(2020)
3	rs4927850	196024759	A	2 x 10-7	1.24	NR	TFRC	22158540(2011)
4	rs6537481	147474942	A	4 x 10-6	1.11	0.75	PRMT5P1, EDNRA	30541042(2019),29422604(2018)
4	rs77962525	8106216	T	3 x 10-7	10.66	NR	ABLIM2	32887889(2020)
5	rs401681	1321972	T	9 x 10-17	1.19	0.44	CLPTM1L	29422604(2018),26098869(2015), 20101243(2010)
5	rs35226131	1295258	C	2 x 10-8	1.49	0.97	TERT, MIR4457	27579533(2016),29422604(2018)
5	rs2736098	1293971	C	7 x 10-15	1.19	0.73	TERT	25086665(2014),29422604(2018)

CHR	SNP	BP	Risk Allele	P value	OR	RAF	Mapped gene	Reference: PubMed ID
5	rs2735948	1299098	A	2 x 10-8	1.45	NR	MIR4457, TERT	32887889(2020)
5	rs6879627	2109787	G	8.16 x 10-6	1.25	0.52	Y_RNA, CTD-2194D22.4	20686608(2010)
5	rs2255280	39394887	T	4 x 10-10	1.23	NR	DAB2	22158540(2011)
6	rs9502893	1339954	G	3.30 x 10-7	1.29	0.35	FOXF2-DT, LINC01394	20686608(2010)
6	rs3016539	161815043	A	1.67 x 10-5	1.42	0.87	PRKN	20686608(2010)
6	rs4269383	155876368	C	7 x 10-7	1.20	NR	RNU7-152P, MIR1202	22158540(2011)
6	rs9363918	68432116	A	1 x 10-6	1.27	NR	LINC02549, ADGRB3-DT	22158540(2011)
7	rs73328514	47448971	A	1 x 10-7	1.18	0.88	TNS3	29422604(2018)
7	rs6971499	130995762	T	7 x 10-14	1.23	0.84	LINC-PINT	25086665(2014),29422604(2018), 26098869(2015)
7	rs17688601	40827064	C	1 x 10-8	1.14	0.73	SUGCT	29422604(2018),26098869(2015)
7	rs4626538	129773348	G	5 x 10-6	0.93	0.49	MIR182, MIR96	34926279(2021)
7	rs6464375	153928758	A	4 x 10-7	3.73	0.10	DPP6	20686608(2010)
7	rs8940	116506020	G	3 x 10-6	2.72	NR	CAV2	33975060(2020)
8	rs2941471	75558169	A	7 x 10-10	1.12	0.57	HNF4G	29422604(2018)
8	rs10094872	127707639	T	1 x 10-9	1.14	0.36	CASC11	27579533(2016),29422604(2018)
8	rs1561927	128555832	T	7 x 10-8	1.12	0.74	LINC00824	25086665(2014),29422604(2018)
8	rs7008921	5566886	C	8 x 10-6	1.42	0.09	RN7SKP159, RPL23AP54	34926279(2021)
8	rs10088262	123753462	A	3.42 x 10-3	1.16	0.34	ANXA13, FAM91A1	20686608(2010)
8	rs7832232	38611785	A	5 x 10-6	1.45	0.45	RNF5P1 , TACC1	20686608(2010)
8	rs2242241	21909370	G	4 x 10-9	1.85	0.01	DOK2	30206226(2018)
9	rs2417487	104125300	A	1.49 x 10-7	1.11	0.44	SMC2	29422604(2018)
9	rs505922	133273813	C	7 x 10-27	1.27	0.35	ABO	29422604(2018),26098869(2015), 19648918
9	rs10991043	104035107	C	7 x 10-6	1.09	0.36	RNA5SP291, SMC2-DT	29422604(2018)
9	rs7859034	104103411	G	3 x 10-7	1.11	NR	SMC2	30541042(2019)
9	rs10974531	4426631	A	5 x 10-6	1.24	NR	RNU6-694P, SLC1A1	22158540(2011)
10	rs12413624	118519432	T	5 x 10-11	1.23	0.37	LINC00867,SLC25A18P1	22158540(2011)

CHR	SNP	BP	Risk Allele	P value	OR	RAF	Mapped gene	Reference: PubMed ID
11	rs145695688	65271879	T	6 x 10-7	2.71	0.01	POLA2	32887889(2020)
11	rs28884829	130754476	A	6 x 10-7	2.29	0.02	PPP1R10P1, LINC02873	32887889(2020)
12	rs1182933	121016819	T	3 x 10-7	1.11	0.30	OASL, C12orf43	29422604(2018)
12	rs7310409	120987058	A	6 x 10-7	1.11	0.39	HNF1A	26098869(2015)
12	rs1169296	120990604	G	3 x 10-7	1.12	NR	HNF1A	30541042(2019)
12	rs789744	64091580	A	4 x 10-6	0.90	0.25	SRGAP1	34216462(2021)
12	rs2035875	52902133	A	7 x 10-10	1.11	0.39	KRT8	34216462(2021)
12	rs708224	32283475	A	3.30 x 10-7	1.32	0.66	BICD1	20686608(2010)
13	rs9581943	27919860	A	2 x 10-9	1.15	0.41	PLUT, PDX1	25086665(2014),29422604(2018), 26098869(2015)
13	rs9543325	73342491	C	1 x 10-22	1.24	0.37	RNY1P8, MARK2P12	34290314(2019),29422604(2018), 26098869(2015),25086665(2014), 20101243(2010)
13	rs9554197	27902841	T	9 x 10-11	1.14	0.40	PLUT	26098869(2015)
13	rs1886449	73357977	A	9 x 10-6	1.21	0.38	MARK2P12	20686608(2010)
13	rs2039553	79725587	A	7 x 10-6	1.73	0.27	LINC01068, LINC01038	20686608(2010)
13	rs1585440	65907683	C	9.28 x 10-6	1.30	0.71	LINC01052	20686608(2010)
13	rs9573163	73334709	G	5 x 10-13	1.26	0.43	RNY1P8, MARK2P12	22158540(2011)
16	rs7200646	86301745	C	1.4 x 10-7	1.12	0.34	LINC00917, LINC01081	29422604(2018)
16	rs7190458	75229763	A	1 x 10-11	1.36	0.04	BCAR1	25086665(2014),29422604(2018)
17	rs77038344	40487962	T	1 x 10-6	1.17	0.08	TNS4	29422604(2018)
17	rs4795218	37718512	G	1 x 10-8	1.14	0.77	HNF1B	29422604(2018)
17	rs7214041	72405335	T	9 x 10-15	1.25	0.11	LINC00511	29422604(2018)
17	rs144239147	50077304	A	5 x 10-7	2.29	0.02	ITGA3	32887889(2020)
17	rs11655237	72404025	T	1 x 10-14	1.26	0.11	LINC00511	26098869(2015)
17	rs876493	39668292	G	1 x 10-6	1.11	NR	PNMT	30541042(2019)
17	rs12951345	37717865	A	1 x 10-6	1.14	NR	HNF1B	30541042(2019)
17	rs7225411	40463878	C	7 x 10-6	1.16	NR	IGFBP4, TNS4	30541042(2019)
17	rs8078692	40058864	A	7 x 10-6	1.10	NR	THRA, MED24	30541042(2019)
17	rs147904962	45377171	A	3 x 10-6	0.75	0.11	ARHGAP27, RNA5SP443	34926279(2021)
17	rs4924935	18850557	G	8 x 10-6	1.37	0.23	PRPSAP2	20686608(2010)

CHR	SNP	BP	Risk Allele	P value	OR	RAF	Mapped gene	Reference: PubMed ID
17	rs225190	32550640	G	5.99 x 10-6	1.26	0.36	MYO1D	20686608(2010)
17	rs2257205	58370936	A	8 x 10-6	1.38	0.33	TSPOAP1-AS1, RNF43	20686608(2010)
18	rs1517037	59211042	C	3 x 10-8	1.16	0.82	GRP, SEC11C	29422604(2018),26098869(2015)
18	rs57791062	59212979	C	2 x 10-6	1.14	NR	SEC11C, GRP	30541042(2019)
19	rs543259829	56301984	G	3 x 10-8	NR	0.001	EDDM13, ZSCAN5A	34594039(2021)
19	rs66562280	21650212	T	3 x 10-8	1.55	0.32	MTDHP3, ZNF429	32671597(2021)
19	rs2656937	4892075	C	1 x 10-7	1.53	0.34	ARRDC5	32671597(2021)
19	rs34309238	14464085	A	5 x 10-10	1.77	0.02	PKN1	30206226(2018)
20	rs6073450	44458008	A	5 x 10-6	1.09	0.37	LINC01620	29422604(2018),26098869(2015)
20	rs76974703	58203411	A	2 x 10-8	4.70	NR	C20orf85, ANKRD60	32887889(2020)
21	rs372883	29345416	T	2 x 10-13	1.27	NR	BACH1	22158540(2011)
21	rs1547374	42358786	A	4 x 10-13	1.27	NR	TFF1, TFF2	22158540(2011)
22	rs450960	17833538	T	7.71 x 10-7	1.11	0.3	MICAL3	29422604(2018)
22	rs16986825	28904318	T	1 x 10-8	1.15	0.15	ZNRF3	25086665(2014),29422604(2018)
22	rs5768709	48533757	G	1 x 10-10	1.25	0.23	TAFA5	22158540(2011)

CHR: The chromosome in which the SNP resides, SNP: Single Nucleotide polymorphism ID, BP: Chromosomal base pair location of the SNP, RAF: Risk allele frequency, OR: Odds ratio, The effect size estimate of the SNP.

Table S4 40 single nucleotide polymorphisms (SNPs) used in calculating the PaCa polygenic risk scores (PRS) in the UK Biobank study.

CHR	SNP	BP	Risk Allele	P value	OR	RAF	Mapped gene	PubMedID
1	rs351365	112503773	C	6 x 10-6	1.11	0.74	WNT2B	29422604(2018)
2	rs1486134	67412637	G	4.61 x 10-9	1.13	0.28	ETAA1, LINC02831	29422604(2018),26098869(2015)
2	rs192904742	6581986	A	9 x 10-7	4.12	NR	MIR7515HG, LINC01824	32887889(2020)
2	rs71411601	52785787	C	2 x 10-7	2.79	0.01	MIR4431, ASB3	32887889(2020)
2	rs962856	67366671	C	2 x 10-8	1.12	0.38	LINC01829	26098869(2015)
3	rs9854771	189790682	G	5 x 10-8	1.11	0.64	TP63	29422604(2018),26098869(2015)
3	rs148512905	18943255	C	1 x 10-7	2.68	0.01	RNU6-138P, SATB1-AS1	32887889(2020)
3	rs138585571	98469736	T	3 x 10-7	1.81	0.04	OR5K1	32887889(2020)
4	rs6537481	147474942	A	4 x 10-6	1.11	0.75	PRMT5P1, EDNRA	30541042(2019),29422604(2018)
4	rs77962525	8106216	T	3 x 10-7	10.66	NR	ABLIM2	32887889(2020)
5	rs401681	1321972	T	9 x 10-17	1.19	0.44	CLPTM1L	29422604(2018),26098869(2015), 20101243(2010)
5	rs35226131	1295258	C	2 x 10-8	1.49	0.97	TERT, MIR4457	27579533(2016),29422604(2018)
5	rs2736098	1293971	C	7 x 10-15	1.19	0.73	TERT	25086665(2014),29422604(2018)
5	rs2735948	1299098	A	2 x 10-8	1.45	NR	MIR4457, TERT	32887889(2020)
7	rs73328514	47448971	A	1 x 10-7	1.18	0.88	TNS3	29422604(2018)
7	rs6971499	130995762	T	7 x 10-14	1.23	0.84	LINC-PINT	25086665(2014),29422604(2018), 26098869(2015)
7	rs17688601	40827064	C	1 x 10-8	1.14	0.73	SUGCT	29422604(2018),26098869(2015)
7	rs4626538	129773348	G	5 x 10-6	0.93	0.49	MIR182, MIR96	34926279(2021)
8	rs10094872	127707639	T	1 x 10-9	1.14	0.36	CASC11	27579533(2016),29422604(2018)
9	rs505922	133273813	C	7 x 10-27	1.27	0.35	ABO	29422604(2018),26098869(2015), 19648918
9	rs10991043	104035107	C	7 x 10-6	1.09	0.36	RNA5SP291, SMC2-DT	29422604(2018)
9	rs7859034	104103411	G	3 x 10-7	1.11	NR	LINC00867,SMC2	30541042(2019)
11	rs145695688	65271879	T	6 x 10-7	2.71	0.01	POLA2	32887889(2020)
11	rs28884829	130754476	A	6 x 10-7	2.29	0.02	PPP1R10P1, LINC02873	32887889(2020)
12	rs7310409	120987058	A	6 x 10-7	1.11	0.39	HNF1A	26098869(2015)
12	rs1169296	120990604	G	3 x 10-7	1.12	NR	HNF1A	30541042(2019)
12	rs2035875	52902133	A	7 x 10-10	1.11	0.39	KRT8	34216462(2021)

CHR	SNP	BP	Risk Allele	P value	OR	RAF	Mapped gene	PubMedID
13	rs9554197	27902841	T	9 x 10-11	1.14	0.40	PLUT	26098869(2015)
16	rs7200646	86301745	C	1.4 x 10-7	1.12	0.34	LINC00917, LINC01081	29422604(2018)
16	rs7190458	75229763	A	1 x 10-11	1.36	0.04	BCAR1	25086665(2014),29422604(2018)
17	rs77038344	40487962	T	1 x 10-6	1.17	0.08	TNS4	29422604(2018)
17	rs4795218	37718512	G	1 x 10-8	1.14	0.77	HNF1B	29422604(2018)
17	rs144239147	50077304	A	5 x 10-7	2.29	0.02	ITGA3	32887889(2020)
17	rs11655237	72404025	T	1 x 10-14	1.26	0.11	LINC00511	26098869(2015)
17	rs12951345	37717865	A	1 x 10-6	1.14	NR	HNF1B	30541042(2019)
17	rs8078692	40058864	A	7 x 10-6	1.10	NR	THRA, MED24	30541042(2019)
17	rs147904962	45377171	A	3 x 10-6	0.75	0.11	ARHGAP27, RNA5SP443	34926279(2021)
18	rs1517037	59211042	C	3 x 10-8	1.16	0.82	GRP, SEC11C	29422604(2018),26098869(2015)
22	rs450960	17833538	T	7.71 x 10-7	1.11	0.30	MICAL3	29422604(2018)
22	rs16986825	28904318	T	1 x 10-8	1.15	0.15	ZNRF3	25086665(2014),29422604(2018)

CHR: The chromosome in which the SNP resides, SNP: Single Nucleotide polymorphism ID, BP: Chromosomal base pair location of the SNP, RAF: Risk allele frequency, OR: Odds ratio, The effect size estimate of the SNP.

Table S5. Missing Rate of four variables.

Variables	Missing numbers	Missing Rate (total n = 258308)	After imputation
**Blood type**	327	0.12%	258,308 (100%)
**WHR**	457	0.17%	258,308 (100%)
**Smoking**	834	0.32%	258,308 (100%)
**Physical activity**	48294	18.69%	258,308 (100%)

Table S6. Demographic characteristics of pancreatic cancer (PaCa) were analyzed across different case and cancer-free control ratio groups.

**Characteristic variables**	**Cases: Controls= 1:10**			**Cases: Controls= 1:15**			**Cases: Controls= 1:20**		
	**Pancreatic cancer (PaCa) cases (n=960)**	**Cancer-free controls (n=9,600)**	**P-value***	**Pancreatic cancer (PaCa) cases (n=960)**	**Cancer-free controls(n=14,400)**	**P-value***	**Pancreatic cancer (PaCa) cases (n=960)**	**Cancer-free controls (n=19,200)**	**P-value***
**Gender**			0.001			0.001			0.001
Female	442 (46.04 %)	4,957 (51.64 %)		442 (46.04 %)	7,419 (51.52 %)		442 (46.04 %)	9,870 (51.41 %)	
Male	518 (53.96 %)	4,643 (48.36 %)		518 (53.96 %)	6,981 (48.48 %)		518 (53.96 %)	9,330 (48.59 %)	
**Age**#	61.60	56.19	<0.001	61.60	56.12	<0.001	61.60	56.08	<0.001
**Polygenic Score (PRS) #(con)**			<0.001			<0.001			<0.001
Standardize PRS	6.12	5.84		6.12	5.83		6.12	5.84	
**Polygenic Score (PRS)(cat)**			<0.001			<0.001			<0.001
Q1	143 (14.90 %)	1,920 (20 %)		137 (14.27 %)	2,880 (20 %)		140 (14.58 %)	3,840 (20 %)	
Q2	135 (14.06 %)	1,920 (20 %)		139 (14.48 %)	2,880 (20 %)		137 (14.27 %)	3,840 (20 %)	
Q3	166 (17.29 %)	1,920 (20 %)		168 (17.50 %)	2,880 (20 %)		167 (17.40 %)	3,840 (20 %)	
Q4	232 (24.17 %)	1,920 (20 %)		232 (24.17 %)	2,880 (20 %)		233 (24.27 %)	3,840 (20 %)	
Q5	284 (960 %)	1,920 (20 %)		284 (29.58 %)	2,880 (20 %)		283 (29.48 %)	3,840 (20 %)	
**Blood type**			<0.001			<0.001			<0.001
O blood type	355 (36.98%)	4,166 (43.40%)		355 (36.98%)	6,235 (43.30 %)		355 (36.98%)	8,340 (43.44 %)	
Non-O blood type	605 (63.02%)	5,434 (56.60%)		605 (63.02%)	8,165 (56.70 %)		605 (63.02%)	10,860 (56.56 %)	
Family history of bowel cancer			0.051			0.101			0.148
No	858 (89.38%)	8,761 (91.26 %)		858 (89.38%)	13,097 (90.95 %)		858 (89.38%)	17,427 (90.77 %)	
Yes	102 (10.63%)	839 (8.74 %)		102 (10.63%)	1,303 (9.05 %)		102 (10.63%)	1,773 (9.23 %)	
**Tobacco smoking status**			<0.001			<0.001			<0.001
Never	441 (45.94%)	5,379 (56.03 %)		441 (45.94%)	8,048 (55.89 %)		441 (45.94%)	10,681 (55.63 %)	
Previous	373 (38.85%)	3,274 (34.10 %)		373 (38.85%)	4,893 (33.98 %)		373 (38.85%)	6,603 (34.39 %)	
Current	146 (15.21%)	947 (9.86 %)		146 (15.21%)	1,459 (10.13%)		146 (15.21%)	1,916 (9.98 %)	
**Alcohol consumption amount**			0.023			0.058			0.094
Never	226 (23.54 %)	2,104 (21.92 %)		226 (23.54 %)	3,184 (22.11 %)		226 (23.54 %)	4,295 (22.37 %)	

Characteristic variables	Cases: Controls= 1:10			Cases: Controls= 1:15			Cases: Controls= 1:20		
	**Pancreatic cancer (PaCa) cases (n=960)**	**Cancer-free controls (n=9,600)**	**P-value***	**Pancreatic cancer (PaCa) cases (n=960)**	**Cancer-free controls(n=14,400)**	**P-value***	**Pancreatic cancer (PaCa) cases (n=960)**	**Cancer-free controls (n=19,200)**	**P-value***
Men: >0-≤28 g/d, Women: >0-≤14 g/d	347 (36.15%)	3,907 (40.70 %)		347 (36.15%)	5,766 (40.04 %)		347 (36.15%)	7,614 (39.66 %)	
Men: >28 g/d, Women: >14 g/d	387 (40.31%)	3,589 (37.39 %)		387 (40.31%)	5,450 (37.85 %)		387 (40.31%)	7,291 (37.97 %)	
**Physical activity (MET-min/week) a**			0.014			0.012			0.008
<600	206 (21.46%)	1,778 (18.52 %)		206 (21.46%)	2,675 (18.58 %)		206 (21.46%)	3,542 (18.45 %)	
600-3000	444 (46.25%)	4,887 (50.91 %)		444 (46.25%)	7,334 (50.93 %)		444 (46.25%)	9,811 (51.10 %)	
>3000	310 (32.29%)	2,935 (30.57 %)		310 (32.29%)	4,391 (30.49 %)		310 (32.29%)	5,847 (30.45 %)	
**BMI**			<0.001			<0.001			<0.001
Normal or Underweight (BMI<25)	249 (25.94%)	3,086 (32.15 %)		249 (25.94%)	4,636 (32.19 %)		249 (25.94%)	6,231 (32.45 %)	
Overweight (25 ≤ BMI < 30)	421 (43.85%)	4,160 (43.33 %)		421 (43.85%)	6,261 (43.48 %)		421 (43.85%)	8,280 (43.13 %)	
Obese (BMI ≥ 30)	290 (30.21%)	2,354 (24.52 %)		290 (30.21%)	3,503 (24.33 %)		290 (30.21%)	4,689 (24.42 %)	
**Waist Hip Ratio (WHR)**			<0.001			<0.001			<0.001
Normal (M: <0.90, F: <0.85)	372 (38.75%)	4,864 (50.67 %)		372 (38.75%)	7,298 (50.68 %)		372 (38.75%)	9,727 (50.66 %)	
Abdominal obesity (M: ≥0.90, F: ≥0.85)	588 (61.25%)	4,736 (49.33 %)		588 (61.25%)	7,102 (49.32 %)		588 (61.25%)	9,473 (49.34 %)	
**Pancreatitis**			<0.001			<0.001			<0.001
No	888 (92.05%)	9,497 (98.93 %)		888 (92.05%)	14,257 (99.01 %)		888 (92.05%)	19,011 (99.02 %)	
Yes	72 (7.5%)	103 (1.07 %)		72 (7.5%)	143 (0.99 %)		72 (7.5%)	189 (0.98 %)	
**Diabetes Mellitus**			<0.001			<0.001			<0.001
No	713 (74.27%)	8,811 (91.78 %)		713 (74.27%)	13,173 (91.48 %)		713 (74.27%)	17,597 (91.65 %)	
Yes	247 (25.73%)	789 (8.22 %)		247 (25.73%)	1,227 (8.52 %)		247 (25.73%)	1,603 (8.35 %)	
**Hepatitis B**			0.479			0.438			0.371
No	960 (100%)	9,595 (99.95 %)		960 (100%)	14,391 (99.94 %)		960 (100%)	19,184(99.92%)	
Yes	0 (0%)	5 (0.05 %)		0 (0%)	9 (0.06 %)		0 (0%)	16 (0.08 %)	

Characteristic variables	Cases: Controls= 1:10			Cases: Controls= 1:15			Cases: Controls= 1:20		
	Pancreatic cancer (PaCa) cases (n=960)	Cancer-free controls (n=9,600)	P-value*	Pancreatic cancer (PaCa) cases (n=960)	Cancer-free controls(n=14,400)	P-value*	Pancreatic cancer (PaCa) cases (n=960)	Cancer-free controls (n=19,200)	P-value*
**Cholecystitis/Cholelithiasis/Cholecystectomy**			<0.001			<0.001			<0.001
No	761 (79.27%)	8,867 (92.36 %)		761 (79.27%)	13,279 (92.22 %)		761 (79.27%)	17,716 (92.27 %)	
Yes	199 (20.73%)	733 (7.64 %)		199 (20.73%)	1,121 (7.78 %)		199 (20.73%)	1,484 (7.73 %)	
**Helicobacter Pylori Infection**			0.299			0.308			0.257
No	949 (98.85%)	9,521 (99.18 %)		949 (98.85%)	14,280 (99.17 %)		949 (98.85%)	19,045 (99.19 %)	
Yes	11 (1.15%)	79 (0.82)		11 (1.15%)	120 (0.83 %)		11 (1.15%)	155 (0.81 %)	
**Systemic Lupus Erythematosus (SLE)**			0.602			0.642			0.556
No	959 (99.90%)	9,583 (99.82 %)		959 (99.90%)	14,376 (99.83 %)		959 (99.90%)	19,164 (99.81 %)	
Yes	1 (0.10%)	17 (0.18 %)		1 (0.10%)	24 (0.17 %)		1 (0.10%)	36 (0.19 %)	
**Vitamin D Deficiency**			0.292			0.324			0.286
No	951 (99.06%)	9,538 (99.35 %)		951 (99.06%)	14,304 (99.33 %)		951 (99.06%)	19,075 (99.35 %)	
Yes	9 (0.94%)	62 (0.65 %)		9 (0.94%)	96 (0.67 %)		9 (0.94%)	125 (0.65 %)	
**Peritonitis**			0.187			0.167			0.190
No	959 (99.90%)	9,565 (99.64 %)		959 (99.90%)	14,345 (99.62 %)		959 (99.90%)	19,131 (99.64 %)	
Yes	1(0.10%)	35 (0.36 %)		1(0.10%)	55 (0.38 %)		1(0.10%)	69 (0.36 %)	

Table S7. Confusion matrix by 10-fold cross-validation method in the training dataset and testing dataset.

	Prediction	ROC	Accuracy
In 85% training dataset	0.996	0.879	0.996
In 15% testing dataset	0.996	0.773	0.996
In 70% training dataset	0.996	0.869	0.996
In 30% testing dataset	0.996	0.749	0.996

Table S8. Pancreatic cancer (PaCa) risk factors in the multivariable logistic regression model across different case and cancer-free control ratio groups.

Characteristic variables	Cases(n=960): Controls(n=9,600) = 1:10			Cases(n=960): Controls(n=14,400) = 1:15			Cases(n=960): Controls(n=19,200) = 1:20		
	**OR**	**95% CI**	**P-value**	**OR**	**95% CI**	**P-value**	**OR**	**95% CI**	**P-value**
**Gender**									
Female	Ref.			Ref.			Ref.		
Male	1.16	(1.01- 1.34)	0.032	1.17	(1.02-1.35)	0.025	1.16	(1.01-1.34)	0.030
**Age**	1.11	(1.09-1.12)	<0.001	1.11	(1.09-1.12)	<0.001	1.11	(1.09-1.12)	<0.001
**Blood type**									
O blood type	Ref.			Ref.			Ref.		
Non-O blood type	1.17	(1.01-1.36)	0.035	1.17	(1.01-1.35)	0.032	1.20	(1.04-0.38)	0.012
**Polygenic Score (PRS)**									
Q1	Ref.			Ref.			Ref.		
Q2	0.94	(0.73- 1.21)	0.627	1.03	(0.80-1.32)	0.830	0.97	(0.76-1.24)	0.820
Q3	1.14	(0.89-1.45)	0.299	1.15	(0.91-1.47)	0.245	1.13	(0.89-1.43)	0.98
Q4	1.57	(1.25-1.98)	<0.001	1.60	(1.24-2.01)	<0.001	1.58	(1.26-1.97)	<0.001
Q5	1.99	(1.59-2.49)	<0.001	2.03	(1.63-2.53)	<0.001	2.0	(1.60-2.47)	<0.001
**Tobacco smoking status**									
Never	Ref.			Ref.			Ref.		
Previous	0.99	(0.85-1.16)	0.941	0.98	(0.84-1.14)	0.771	0.96	(0.83-1.12)	0.630
Current	1.82	(1.47-2.26)	<0.001	1.73	(1.40-2.13)	<0.001	1.78	(1.45-2.18)	<0.001
**Alcohol consumption amount**									
Never	Ref.			Ref.			Ref.		
Men: >0-≤28 g/d, Women: >0-≤14 g/d	0.90	(0.75-1.09)	0.303	0.93	(0.77-1.12)	0.456	0.97	(0.81-1.16)	0.742
Men: >28 g/d, Women: >14 g/d	1.25	(1.03-1.51)	0.021	1.26	(1.05-1.52)	0.013	1.30	(1.08-1.56)	0.005
**Pancreatitis**									
No	Ref.			Ref.			Ref.		
Yes	4.02	(2.85-5.68)	<0.001	4.37	(3.16-6.04)	<0.001	4.28	(3.13-5.85)	<0.001
**Diabetes Mellitus**									
No	Ref.			Ref.			Ref.		
Yes	2.64	(2.21-3.15)	<0.001	2.51	(3.16-6.04)	<0.001	2.61	(2.21-3.08)	<0.001
**Cholecystitis/Cholelithiasis/Cholecystectomy**									
No	Ref.			Ref.			Ref.		
Yes	2.25	(1.85-2.74)	<0.001	2.14	(1.78-2.58)	<0.001	2.14	(1.78-2.57)	<0.001

Table S9. Bootstrap 500 repetitions for internal validation of the model performance.

	**Repetitions**	**Observed**	**Bias**	**Std. err.**	**95% conf. interval**
The area under the receiver operating characteristic curve (ROC) curve	500	0.7762175	0.0019289	0.0070307	0.762-0.790 (N)
					0.765-0.792 (P)
					0.763-0.789 (BC)

N: Normal, P: Percentile, BC: Bias-corrected

Table S10. The equation for constructing the nomogram.

Characteristic variables	Point	Characteristic variables	Point
**Gender**		**Diabetes Mellitus (DM)**	
Female	0	No	0
Male	3.897524	Yes	23.7858
**Age**	points = 0 * Age ^2 + 2.5 * Age + -87.5	**Cholecystitis/Cholelithiasis/Cholecystectomy**	
**Blood type**		No	0
O blood type	0	Yes	17.93196
Non-O blood type	4.351187		
**Polygenic score (PRS)**			
Q1	0		
Q2	1.216108		
Q3	4.967657		
Q4	12.917783		
Q5	17.833972		
**Tobacco smoking status**			
Never	0		
Previous	0.2279377		
Current	15.0792601		
**Alcohol intake amount**			
Never	0		
Men>0-28g/d, Women>0-14 g/d	0.3316719		
Men>28g/d, Women>14 g/d	6.0683227		
**Pancreatitis**			
No	0		
Yes	34.87298		
Predicted Value=2.16e-07 * points ^3 + -5.9989e-05 * points ^2 + 0.005434161 * points + -0.14917944			

502,387 Participants in the UKB cohort , 2006-2010

Exclude:

92 participants defined as pancreatic cancer prevalence cases.

111,866 participants had the history of other neoplasms.

390,429 participants:

1,402 participants were pancreatic cancer incident cases.

389,027 participants were cancer-free controls.

Exclude:

130,719 participants didn’t pass sample QC.

258,308 participants:

960 participants were pancreatic cancer incident cases.

257,348 participants were cancer-free controls.

Figure S1. The flowchart of PaCa cases and cancer-free controls selection process in the UKB cohort.

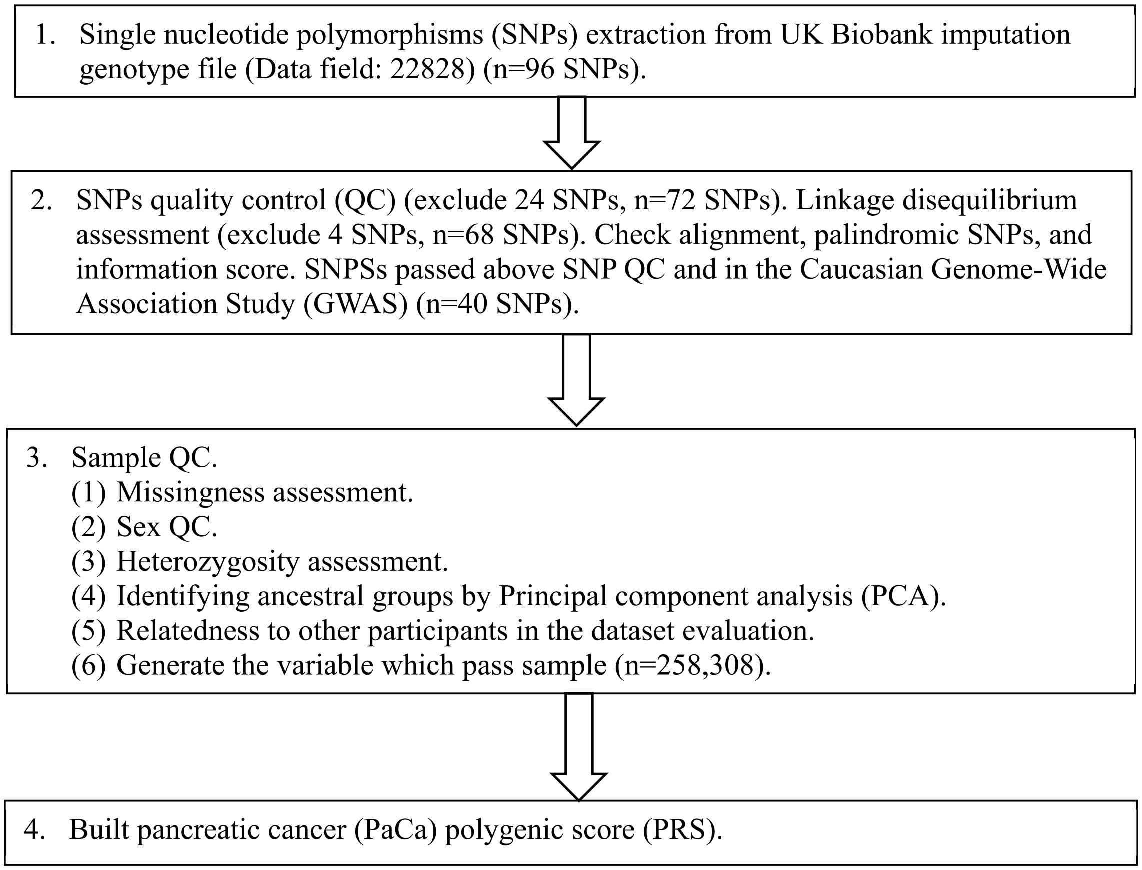

Figure S2 The flowchart of SNPs extraction, SNPs QC, Sample QC and PRS construction.

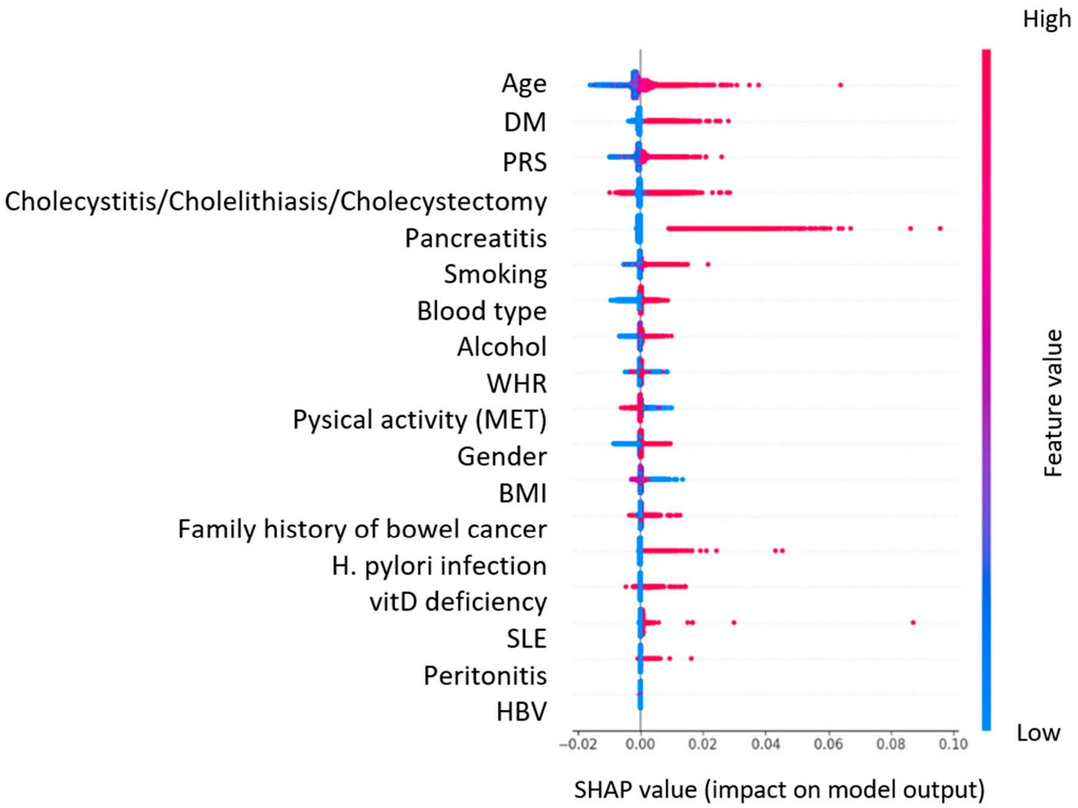

Figure S3. The SHAP summary plot

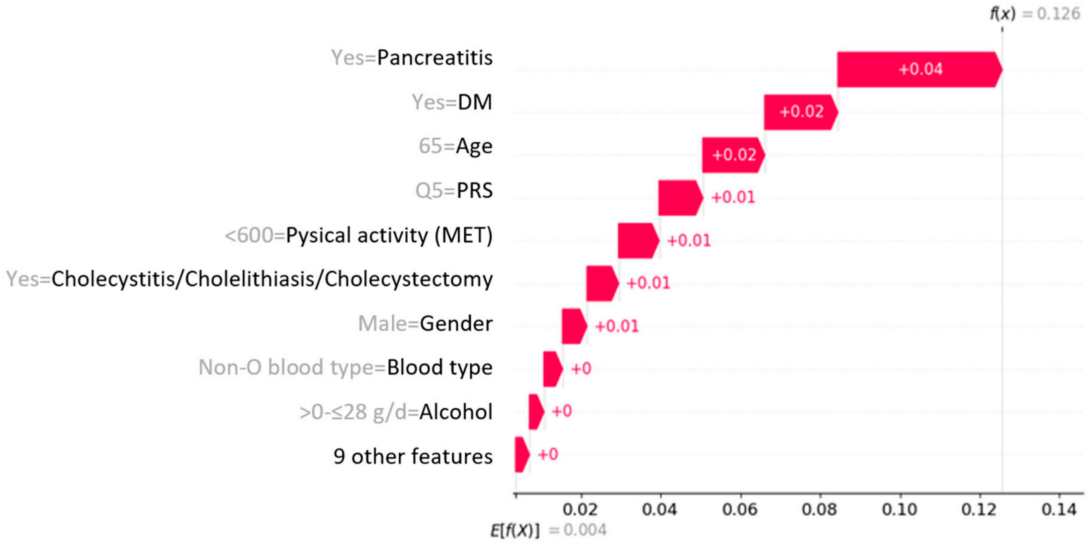

Figure S4. SHAP waterfall plot. A SHAP waterfall plot (Figure S4) was utilized to demonstrate how the risk variables affect pancreatic cancer risk in one of our samples. This plot illustrates how features such as having pancreatitis, diabetes mellitus (DM), being 65 years old, having a Polygenic Risk Score (PRS) of Q5, engaging in <600 MET/week of physical activity, having gallbladder disease, being male, having a non-O blood type, and consuming >0 to ≤ 28 g/day of alcohol) impact the pancreatic cancer risk in this individual within our predictive model.

(a) In the 85% training dataset

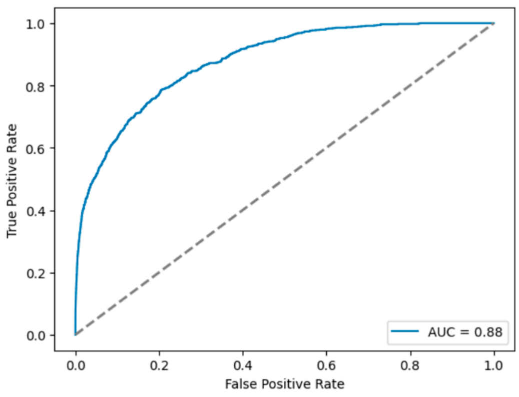

(b) In the 15% testing dataset

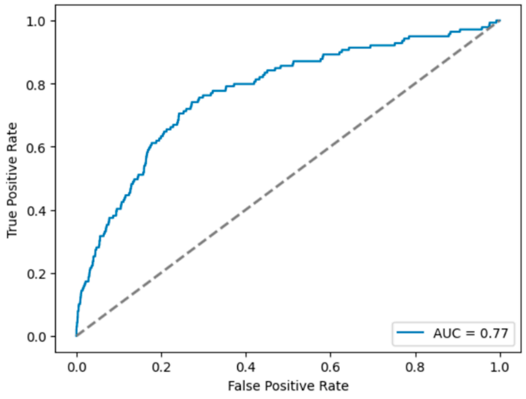

(c) In the 70% training dataset

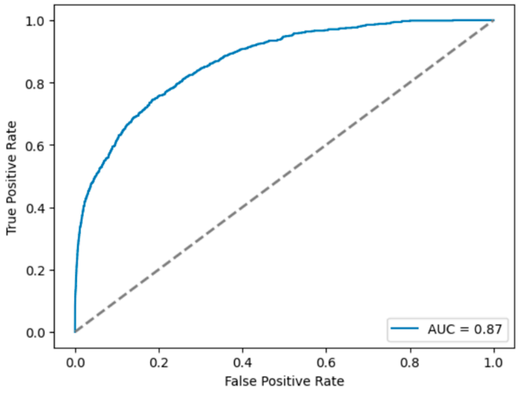

(d) In the 30% testing dataset

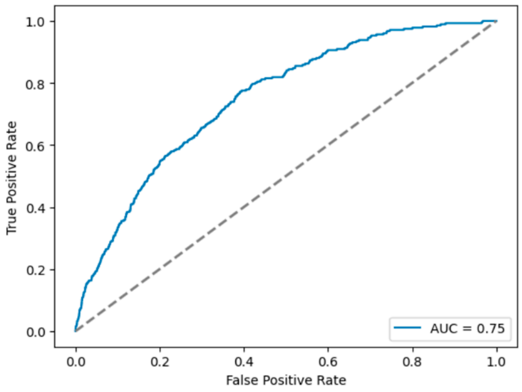

Figure S5. The receiver operating characteristic (ROC) curve for the random forest. (a) The receiver operating characteristic (ROC) curve for the random forest in the 85% training dataset. (b) The receiver operating characteristic (ROC) curve for the random forest in the 15% testing dataset. (c) The receiver operating characteristic (ROC) curve for the random forest in the 70% training dataset. (d) The receiver operating characteristic (ROC) curve for the random forest in the 30% testing dataset.

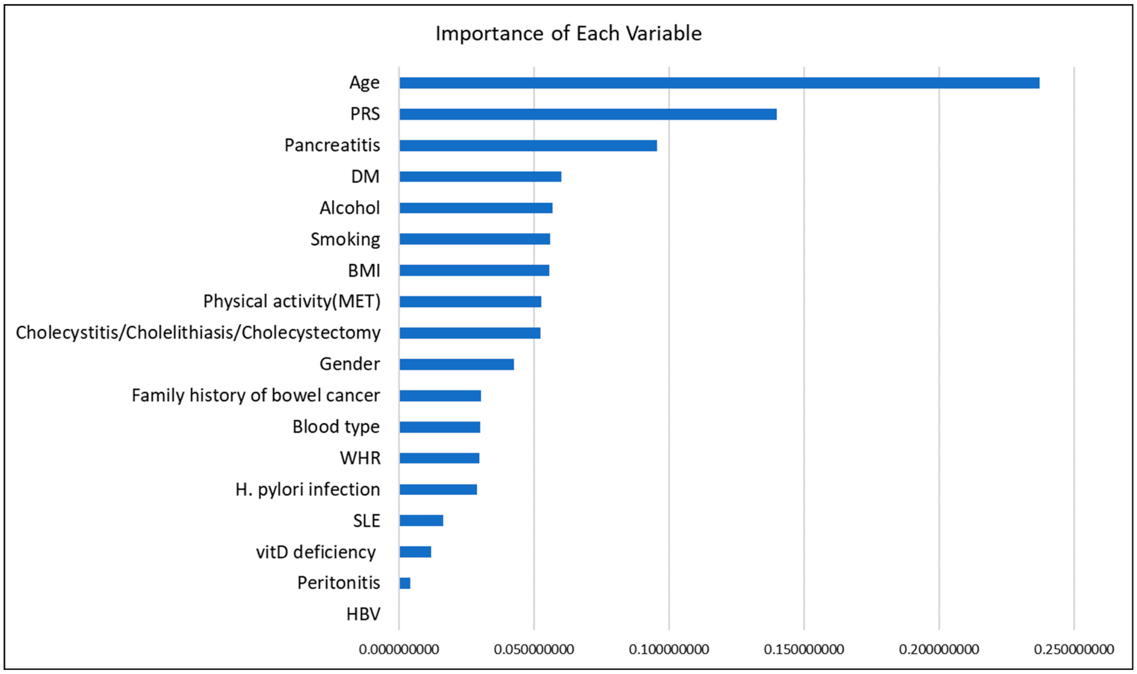

Figure S6. The importance of each variable in the sensitivity test.

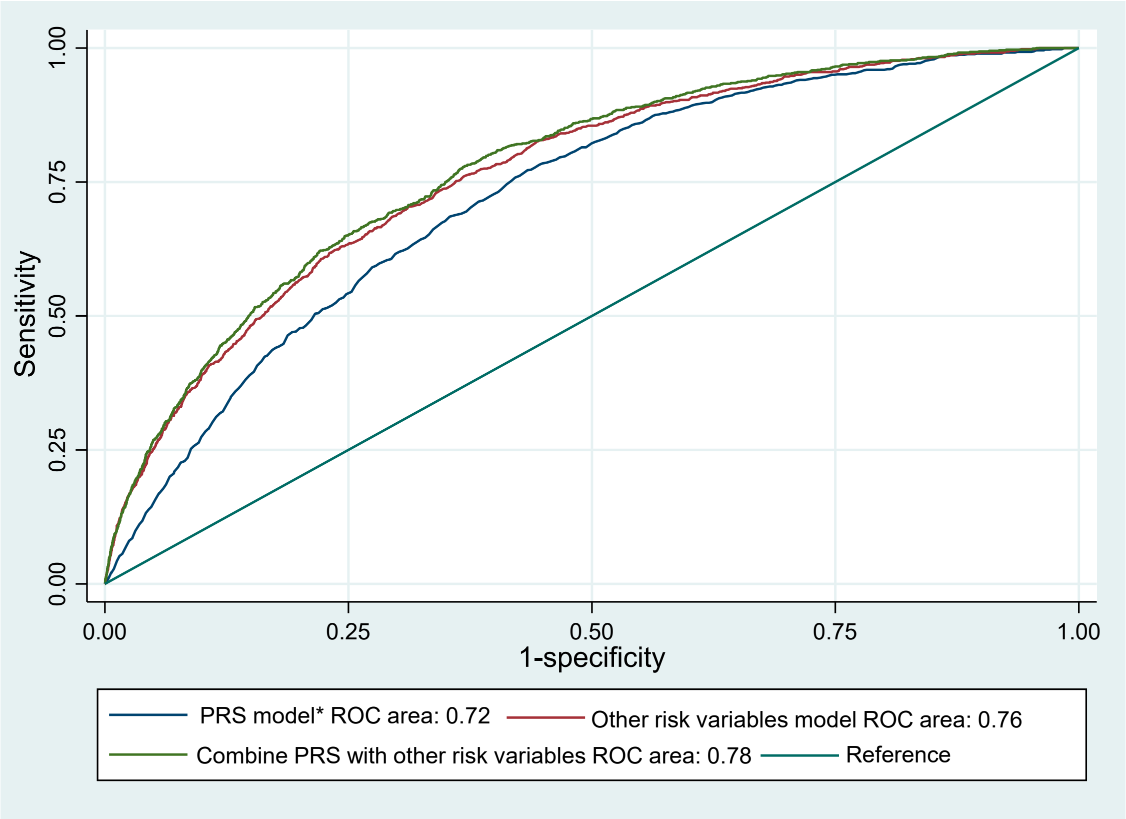

Figure S7. The receiver operating characteristic curve (ROC) for whole logistic regression and without PRS model.

*PRS model was adjusted by gender and age.

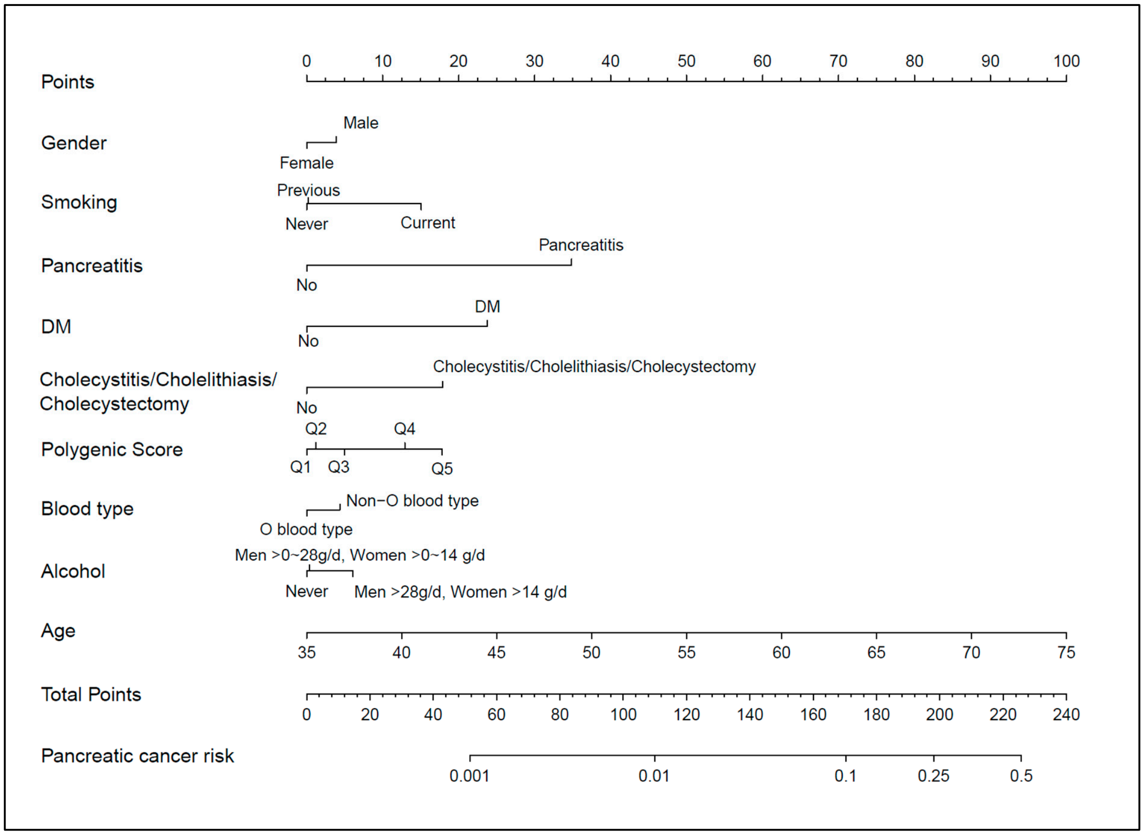

Figure S8. A nomogram based on the multivariable logistic model.

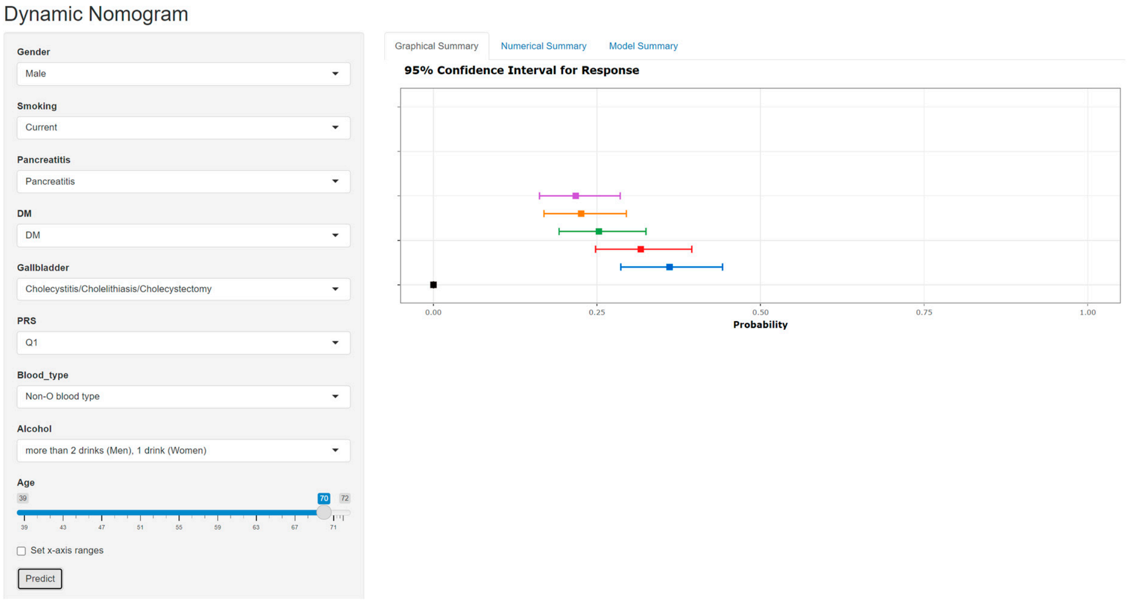

Figure S9. A dynamic nomogram based on the multivariable logistic model. (https://ts35ky-temin-ke.shinyapps.io/DynNomapp/)
